# Supplementary material for: Content-rich biological network constructed by mining PubMed abstracts
Source: BMC Bioinformatics. 2004 Oct 8;5:147. doi: 10.1186/1471-2105-5-147 (PMC528731; doi:10.1186/1471-2105-5-147)
Supplement: Additional File 2 — The original results of the above study (non-essential files are deleted to keep the file size under the limit set by BMC bioinformatics). [file 1471-2105-5-147-S2.bz2 › chilibotAdditionalFile2/dip05/557ID10395669E10917/html/IFNA2_IFNAR2.html]

 


 **IFNA2** and **IFNAR2** 
  
Found 4 abstracts in PubMed, retrieved 4.  
 

 What does Google say? 
 PDF only 
| .edu only 

---

**Interactive relationship** (e.g. stimulation, inhibition, etc)

**Neutral relationship**- We have employed spectroscopic interferometry for label free monitoring of the interaction between the cytokine interferon alpha2  [ **IFNA2** ]  IFNalpha2 and the extracellular domain of its receptor  **ifnar2**   **ifnar2**  EC.  Ref: 11161311 Anal Biochem, 2001
- Here, we report purification and refolding of the extracellular part of human  **ifnar2**   **ifnar2**  EC expressed in Escherichia coli and its characterization with respect to its interaction with interferon alpha2  [ **IFNA2** ]  IFNalpha2.  Ref: 10339405 J Mol Biol, 1999
